# Investigating *Campylobacter* Contamination of Broiler Chicken in Isfahan Province (Iran) and Evaluating the Antibiotic Resistance Patterns of the Isolates

**DOI:** 10.1155/vmi/8261818

**Published:** 2026-06-19

**Authors:** Sajjad Abedini Chamgordani, Mehdi Rezaei, Moslem Neyriz Naghadehi

**Affiliations:** ^1^ Faculty of Veterinary Medicine, Ur.C., Islamic Azad University, Urmia, Iran, azad.ac.ir; ^2^ Department of Clinical Sciences, Faculty of Veterinary Medicine, Ur.C., Islamic Azad University, Urmia, Iran, azad.ac.ir; ^3^ Department of Pathobiology, Faculty of Veterinary Medicine, Ur.C., Islamic Azad University, Urmia, Iran, azad.ac.ir

**Keywords:** antibiogram, broiler chickens, *Campylobacter*, microaerophilic, PCR

## Abstract

Thermophilic *Campylobacter* species are a major cause of bacterial diarrhea transmitted to humans through the consumption of raw or undercooked food derived from livestock and poultry. This study aimed to isolate and identify thermophilic *Campylobacter* strains in broiler chickens from Isfahan Province using culture and polymerase chain reaction (PCR) methods and to determine the antibiotic susceptibility of the isolates. A total of 200 fecal samples were randomly collected from 20 broiler flocks in Isfahan Province between October 2023 and late March 2024. Samples were cultured in specific media under microaerophilic conditions. Molecular identification was based on the detection of an 816‐base pair band (16S rRNA gene—*Campylobacter* genus), a 462‐base pair band (*ceuE* gene—*Campylobacter coli*), and a 589‐base pair band (*mapA* gene—*Campylobacter jejuni*). Of the 200 fecal samples, 4 (2%) were identified as contaminated with *Campylobacter* spp., with 2 (50%) identified as *C. coli* and 2 (50%) as *C. jejuni*. In antibiotic susceptibility tests, all isolates (100%) were resistant to erythromycin, azithromycin, streptomycin, tetracycline, gentamicin, doxycycline, sultrim, chloramphenicol, and ciprofloxacin. *C*. *jejuni* isolates (100%) were susceptible to ceftriaxone, while one *C. coli* isolate (50%) showed intermediate susceptibility. Additionally, one *C. jejuni* isolate (50%) was susceptible to lincomycin–spectinomycin. Moreover, all isolates (100%) exhibited multidrug resistance (MDR). The results of this study reveal the presence of *Campylobacter* contamination in broiler poultry in Isfahan Province, highlighting its role as a zoonotic pathogen. Based on the observed antibiotic resistance patterns, it is recommended to enforce stricter regulations on the use of antibiotics in the poultry industry.

## 1. Introduction

The increasing global consumption of chicken meat has led to a rise in its production compared to other protein sources [1]. Poultry, during production, slaughter, and distribution, may become infected with zoonotic pathogens, potentially transmitting them to humans. The genus *Campylobacter*, belonging to the family Campylobacteraceae, is a curved, S‐shaped, flagellated bacterium characterized by its darting motility. These bacteria can transform into spherical and coccoid forms as a survival response under stress [2]. *Campylobacter* typically grows under microaerophilic conditions and is commonly found in the bodies of mammals and birds [3]. Species such as *Campylobacter fetus* and *Campylobacter jejuni* are responsible for abortions in ruminants, leading to economic losses in the livestock industry [4]. Among documented species [5], *C. jejuni*, *C. coli*, *C. lari*, and *C. hepaticus* usually do not grow at temperatures below 30°C and exhibit optimal growth at 42°C. These are recognized as thermophilic *Campylobacter* species and are significant due to their role in causing diseases in humans and animals [6, 7]. Thermophilic *Campylobacter* are among the most common causes of bacterial diarrhea in humans, primarily spread through poultry, raw milk, and contaminated water [6, 8]. Transmission occurs via the fecal–oral route, and the incidence of the disease in humans is closely linked to the level of contamination of food sources by this bacterium [9, 10]. *Campylobacter* infection in humans may result in fever, watery or bloody diarrhea, abdominal cramps, nausea, vomiting, meningitis, and post‐infection conditions such as Guillain–Barré syndrome and arthritis, which can occasionally be fatal. Global spread, significant diarrheal incidence, association with Guillain–Barré syndrome, and mortality, though occurring at a low rate, are among the reasons why campylobacteriosis is recognized by the WHO as an important zoonotic disease [8, 11, 12]. Although *Campylobacter* organisms exist as part of the normal flora in the gastrointestinal tract of birds and can become pathogenic under stress, they are rarely detected in birds younger than 2‐3 weeks, likely due to maternal immunity [3]. Consequently, contamination in broiler chickens rarely manifests as clinical symptoms [2].

The pathogenicity of *Campylobacter* occurs through the invasion of the epithelial cells of the gastrointestinal tract in birds. *C*. *jejuni* has the capacity to cause intestinal inflammation and diarrhea in specific strains of broiler chickens when studied under controlled laboratory conditions. Historically, studies have reported an association between *C. jejuni* and *C. coli* with vibrionic hepatitis in poultry. Diarrhea and a decrease in egg production are the most common symptoms of this hepatitis in poultry [2, 13, 14]. Currently, vibrionic hepatitis is known as “spotty liver syndrome,” caused by *Campylobacter hepaticus* and, more recently, *Campylobacter bilis*, which induces pathological lesions in the intestine and liver (miliary gray spots) and leads to mortality. It is essential to differentiate this infection from *C. coli* and *C. jejuni* [2, 15, 16]. The prevalence of *Campylobacter* contamination in poultry farms varies significantly, ranging from 0% (*Campylobacter*‐free flocks) to 100% (entirely contaminated flocks) across different flocks [2]. The high ability of bacteria to transfer resistance mechanisms to each other and the rapid rate of this process are among the main reasons for global concern about this issue. The importance of this matter increases when the zoonotic bacterium *Campylobacter* acquires antibiotic resistance, which can make treatment difficult if transmitted to humans. This study investigates thermophilic *Campylobacter* species from broiler flocks in Isfahan Province using both culture and polymerase chain reaction (PCR) methods. Additionally, it assesses the antibiotic resistance patterns of the isolates to not only identify the bacteria but also take an effective step toward appropriate biosecurity measures in controlling and preventing the disease in the region.

## 2. Materials and Methods

### 2.1. Sampling

This descriptive and applied study was conducted from October 2023 to late March 2024. A total of 200 fresh fecal samples were randomly collected from 20 apparently healthy flocks at least 10 days old that followed an all‐in all‐out management system, while flocks with diarrhea or of different ages were excluded from the study. Samples were transported on ice in a 0.1% (w/v) buffered peptone water solution to the reference laboratory within an average time of 4 h.

### 2.2. Microbial Culture

Samples were enriched in selective *Campylobacter* broth (Quelab, Canada) supplemented with Skirrow (Quelab, Canada) and 5% defibrinated sheep blood under microaerophilic conditions (85% nitrogen, 10% carbon dioxide, and 5% oxygen) at 42°C. They were then cultured on selective *Campylobacter* agar (Quelab, Canada), containing defibrinated sheep blood and Skirrow supplement (Quelab, Canada), and incubated under microaerophilic conditions at 42°C. Initial identification was based on phenotypic characteristics: gray colonies, motility, Gram staining, morphology S‐shaped curved, and biochemical tests, including catalase and oxidase tests for the *Campylobacter* genus and a hydrolyzed hippurate (Neutron, Iran) test to differentiate *C. jejuni* and *C. coli* species.

### 2.3. Molecular PCR Test

DNA extraction was performed using a kit from MBST (Iran). To amplify the *mapA* and *ceuE* genes along with 16S rRNA, primers recommended by Inglis and Kalischuk were used [17]. The primer sequences and target genes are outlined in Table 1. For the PCR assay, the main reaction mix (25 μL) was prepared for all test samples. The target fragment was amplified using a master mix from SinaClon (Iran). The thermal cycling program is detailed in Table 2. The PCR product was electrophoresed for 35 min in a 1.5% agarose gel at 120 V and examined under UV light.

**TABLE 1:** Primers used in the PCR test [17].

Gene	Sequence	Product size (bp)	Feature
16S rRNA	GGATGACACTTTTCGGAGC—forward	816	*Campylobacter* spp.
	CATTGTAGCACGTGTGTC—reverse		

*mapA*	CTATTTTATTTTTGAGTGCTTGTG—forward	589	*C. jejuni*
	GCTTTATTTGCCATTTGTTTTATTA—reverse		

*ceuE*	ATTTGAAAATTGCTCCAACTATG—forward	462	*C. coli*
	TGATTTTATTATTTGTAGCAGCG—reverse		

**TABLE 2:** Time, temperature, and the number of cycles in the thermocycler program.

Steps	Bacteria/gene	Temperature (°C)	Time	Number of cycles
Initial denaturation	Spp. (16S rRNA)	94	5 min	Spp. (35), *C. coli* (35), *C. jejuni* (35)
	*C. coli* (*ceuE*)	94	3 min	
	*C. jejuni* (*mapA*)	94	5 min	
Second denaturation	Spp. (16S rRNA)	94	1 min	
	*C. coli* (*ceuE*)	94	1 min	
	*C. jejuni* (*mapA*)	94	45 sec	
Annealing	Spp. (16S rRNA)	57	45 sec	
	*C. coli* (*ceuE*)	52	45 sec	
	*C. jejuni* (*mapA*)	56	1 min	
DNA extension	Spp. (16S rRNA)	72	45 sec	
	*C. coli* (*ceuE*)	72	45 sec	
	*C. jejuni* (*mapA*)	72	1 min	
DNA extension	Spp. (16S rRNA)	72	5 min	
	*C. coli* (*ceuE*)	72	5 min	
	*C. jejuni* (*mapA*)	72	7 min	

**TABLE 3:** The prevalence of MDR *Campylobacter* strains isolated from fecal samples of broiler farms in Isfahan Province (S = sensitive, I.S = intermediate sensitive, and R = resistant).

Antibiotics	*Campylobacter* species (*N = 4*)			*C*. *jejuni* (*N* = 2)			*C. coli* (*N* = 2)		
	S	I.S	R	S	I.S	R	S	I.S	R
Erythromycin	—	—	4 (100%)	—	—	2 (100%)	—	—	2 (100%)
Azithromycin	—	—	4 (100%)	—	—	2 (100%)	—	—	2 (100%)
Streptomycin	—	—	4 (100%)	—	—	2 (100%)	—	—	2 (100%)
Tetracycline	—	—	4 (100%)	—	—	2 (100%)	—	—	2 (100%)
Gentamicin	—	—	4 (100%)	—	—	2 (100%)	—	—	2 (100%)
Doxycycline	—	—	4 (100%)	—	—	2 (100%)	—	—	2 (100%)
Ceftriaxone	2 (50%)	1 (25%)	1 (25%)	2 (100%)	—	—	—	1 (50%)	1 (50%)
Sultrim	—	—	4 (100%)	—	—	2 (100%)	—	—	2 (100%)
Ciprofloxacin	—	—	4 (100%)	—	—	2 (100%)			2 (100%)
Chloramphenicol	—	—	4 (100%)	—	—	2 (100%)			2 (100%)
Tetracycline	—	—	4 (100%)	—	—	2 (100%)	—	—	2 (100%)
Lincospectin	1 (25%)	—	3 (75%)	1 (50%)	—	1 (50%)	—	—	2 (100%)

### 2.4. Antimicrobial Susceptibility

Antimicrobial susceptibility testing was conducted using the Kirby–Bauer disk diffusion method for the following antibiotics: azithromycin (15 μg), gentamicin (10 μg), chloramphenicol (30 μg), tetracycline (30 μg), streptomycin (10 μg), erythromycin (15 μg), lincomycin (2 µg), doxycycline (30 μg), ceftriaxone (30 μg), ciprofloxacin (5 µg), and sultrim (30 μg) (Padtan Teb, Iran). Suspensions prepared based on a 0.5 McFarland standard, containing 1.5 × 10^8^ bacteria, were inoculated on Mueller–Hinton agar plates containing 5% defibrinated sheep blood. Following a 24‐hour incubation period under microaerophilic conditions at 42°C, the diameters of the bacterial growth inhibition zones were measured and interpreted according to the Clinical and Laboratory Standards Institute guidelines [18].

## 3. Results

**FIGURE 1:**
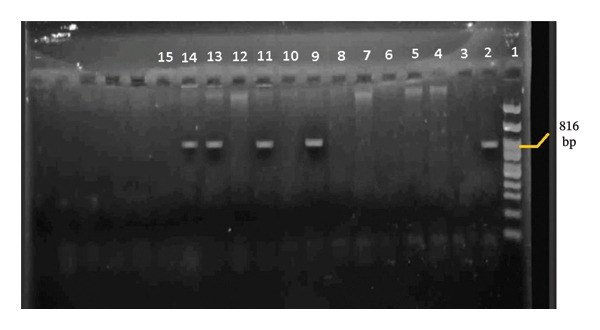
Standard PCR for *Campylobacter* spp. detection targeting 16S rRNA gene (816 bp). Detection of 16S rRNA gene in various samples. Lane 1: 100‐bp DNA ladder; lane 2: positive control; lane 3: negative control; lanes 9, 11, 13, and 14: positive for *Campylobacter* spp.; lanes 4–8, 10, 12, and 15: other tested samples (negative for *Campylobacter* spp.).

**FIGURE 2:**
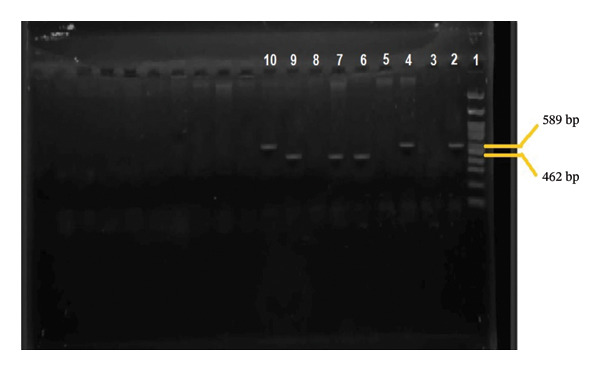
Multiplex PCR for *Campylobacter* species identification targeting ceuE (462 bp) for C. *coli* and mapA (589 bp) for C. *jejuni*. Detection of mapA and ceuE genes in various samples. Lane 1: 100 bp DNA ladder; lanes 2 and 4: positive for C. *jejuni* (589 bp); lanes 6 and 7: positive for C. *coli* (462 bp); lane 8: negative control; lane 9: positive control for C. *coli*; lane 10: positive control for C. *jejuni*.

Out of 200 fecal samples, 12 *Campylobacter* bacteria were initially identified after culturing, microscopic examination, and biochemical tests. These isolates were Gram‐negative, oxidase‐positive, and catalase‐positive. Based on the hippurate hydrolyzed test, 2 isolates were identified as *C. jejuni* (hippurate positive) and 10 as *C. coli* (hippurate negative). Amplification of the 16S rRNA gene fragment on 1.5% agarose gel revealed a band of 816 bp, indicating four PCR‐positive samples (2%) for the *Campylobacter* genus (Figure 1). Among these, two samples (50%) were identified as *C. coli*, with a *ceuE* gene band at 462 bp, and two samples (50%) as *C. jejuni*, with a *mapA* gene band at 589 bp (Figure 2).

The results of the antibiogram test revealed that the isolates were sensitive to ceftriaxone (100% of *C. jejuni* isolates were sensitive, and 50% of *C. coli* isolates were intermediately sensitive) and lincomycin–spectinomycin (50% of *C. jejuni* isolates). All isolates exhibited resistance to erythromycin, azithromycin, streptomycin, tetracycline, gentamicin, doxycycline, sultrim, chloramphenicol, and ciprofloxacin. All isolates were classified as multidrug‐resistant (MDR) (Table 3).

## 4. Discussion

While thermophilic *Campylobacter* species are generally asymptomatic in poultry, they cause diarrhea in humans and are associated with post‐infectious diseases and in consequence negatively affect public health and the economies of countries worldwide. Infected chicken meat plays a major role in the transmission of this bacterium. Improper evisceration in the slaughterhouse may result in cross‐contamination, leading to large‐scale transmission of the pathogen to consumers [2, 15, 16, 19, 20].

The objective of this study was to isolate and molecularly identify thermophilic *Campylobacter* species in fecal samples from broiler chickens using culture, biochemical methods, and PCR, along with determining the antibiotic susceptibility of the isolates. Numerous reports have been published regarding the prevalence of *Campylobacter* worldwide. In the United States, Poudel et al. reported a *Campylobacter* contamination rate of 25.4% based on molecular analysis of samples collected from farms and poultry meat retail [21]. In China, Bai et al. reported a *Campylobacter* contamination rate of 14.2% by examining broiler flocks and slaughter line stages [22]. In Malaysia, Sinulingga et al. identified *Campylobacter* infection in cloacal swabs of broiler chickens (50.9%) and in poultry meat sold at markets (26.6%), finding significantly higher prevalence rates in open rearing systems (46%–86%) compared to closed systems (0%–2%) [23]. Ogbor et al. found 5.3% contamination of *Campylobacter* bacteria from poultry feces in Nigeria, with all identified isolates being *C. coli* and showing MDR [24]. A study in Turkey identified *Campylobacter* contamination in 39.3% of farmed geese using PCR molecular testing, with 96.2% being *C. jejuni* and 3.8% *C. coli* [25].

In Iran, multiple studies have investigated *Campylobacter* prevalence in poultry. Reported contamination rates include 25.09% in Shahrekord [26], 28.9% in Shiraz [27], and 0.6% in Khuzestan Province in chicken meat [28]. No recent data are available on the prevalence of *Campylobacter* in Isfahan Province. Unlike this study, which focuses on identifying *Campylobacter* in fecal samples, previous studies in Isfahan have evaluated *Campylobacter* prevalence in poultry meat. Rahimi and Tajbakhsh reported a prevalence rate of 47.1% in various poultry meats from Isfahan based on biochemical tests [29]. In the present study, the sample size calculation was performed assuming simple random sampling and did not account for the design effect associated with clustering at the flock level. Of the 12 *Campylobacter* samples (6%) initially identified through culture and biochemical methods, only 4 samples (2%) were confirmed via PCR. Among these four isolates identified at the genus level by PCR, two were *C. jejuni* (50%) and two were *C. coli* (50%). The contamination rate identified in this study is notably lower than that reported in many other studies [24, 30]. The observed differences may be attributed to variations in sampling methods, testing techniques, flock density, rearing systems, hygiene and biosecurity measures [31], stress factors, and medication use [23], as well as additives in water, feed, and litter, in addition to geographical location, season, and broiler age [2, 3]. Higher *Campylobacter* prevalence in warmer seasons compared to colder seasons may be due to the increase in insect populations during warmer months [2, 29, 32, 33]. In the current study, this observation was also noted, given that the sampling was conducted during the winter season, resulting in a lower detection rate of the bacterium.

Diarrhea caused by this toxigenic bacterium, although self‐limiting in humans, is notable due to its high prevalence and zoonotic potential. Among *Campylobacter* species, *C. jejuni* is the most pathogenic to humans. Moreover, *C. jejuni* typically predominates among thermophilic species in poultry. However, in certain cases, populations of *C. coli* have been reported to exceed those of *C. jejuni* [20, 28]. The probable cause of this population shift has been attributed to antibiotic pressure [2]. However, this is in contrast with the findings of the present study, where the detection rates of *C. jejuni* and *C. coli* populations were identical. *Campylobacter* is fastidious and may be difficult to isolate in fecal culture due to overlap with other bacteria [34], Since PCR testing detects the pathogen based on its DNA and the viability of the bacteria, whether living or dead, does not affect its ability to identify the causative agent, PCR was used in this study as a supplementary method to confirm the cultural and biochemical diagnosis. Considering that bacteria such as *Pseudomonas*, *E. coli*, *Klebsiella*, *Salmonella*, *Proteus*, and some cocci show similar responses to *Campylobacter* in biochemical tests (e.g., oxidase and catalase tests), they may result in false‐positive results that influence the outcomes of these tests [35–38]. In addition, as red blood cells (RBCs) contain catalase, this could lead to false‐positive catalase test results. Furthermore, certain substances in the culture medium and lab equipment, such as glucose‐containing media and metallic loops, can cause false‐positive results in the oxidase test [39].

The antibiotics ciprofloxacin (fluoroquinolones) and erythromycin (macrolides) are significant in the treatment of human campylobacteriosis [40]. Because *Campylobacter*‐related diarrhea is self‐limiting, rehydration with fluids and electrolytes is sufficient for diarrheal cases, and antibiotic therapy is necessary only for severe cases, such as those involving immunodeficiency. Misuse of antibiotics can lead to antibiotic resistance [8, 41]. Most human antimicrobial agents are also used in the poultry industry in some Asian countries. This issue raises significant concerns about the increase in antibiotic‐resistant strains isolated from poultry [42]. Therefore, the antibiotic resistance patterns in poultry could serve as a guide for treating human cases [2, 25]. The evaluation of antimicrobial sensitivity in the samples collected in this study showed 100% resistance of the isolates to the antibiotics sultrim, ciprofloxacin, erythromycin, azithromycin, tetracycline, doxycycline, streptomycin, and gentamicin. The resistance observed in this study is similar in intensity to that reported by Demiroğlu et al., Fani et al., Mousavinafchi et al., Nshama et al., Ogbor et al., Ommi et al., Poudel et al., Sabzmeydani et al., and Sadeghi et al. [21, 24–27, 30, 33, 43, 44]. In contrast to the current study, the findings of Maktabi et al., Kalantar et al., and Rahimi and Ameri showed 100% susceptibility of the isolates to gentamicin [20, 28, 45]. Meanwhile, Fani et al. (50% in *C. coli*), Mousavinafchi et al. (4.1% in C. *jejuni*), and Sabzmeydani et al. (1.15% in *C. jejuni*) have reported varying percentages of resistance to gentamicin in different species [26, 27, 44]. Maktabi et al. reported 6.2% resistance to erythromycin [28], and Khan et al. reported 6.9% [35], which both show a significant difference from the findings of this study. The differences in study results regarding resistance to gentamicin and erythromycin may be attributed to the diversity of resistance genes on plasmids and their high capacity for horizontal transfer from other microorganisms to *Campylobacter* [46]. Studies by Mousavinafchi et al., Fani et al., and Sadeghi et al. have reported high levels of resistance to tetracycline, which is consistent with the findings of this study [26, 27, 43]. Tetracyclines are frequently prescribed for livestock due to their low cost, high efficacy, and broad‐spectrum activity, which has contributed to the high resistance observed in *Campylobacter* [42].

Of the 12 antibiotics tested in this study, sensitivity was observed only to two antibiotics: lincomycin–spectinomycin (lincospectin) and ceftriaxone (Table 3). A study evaluating the sensitivity of *Campylobacter* to lincospectin was not available, suggesting that this study may be the first assessment of *Campylobacter* isolates’ sensitivity to this antibiotic. Additionally, lincospectin has only recently been introduced for use in poultry. This drug combines two antibiotics, lincomycin and spectinomycin, and probably due to their synergistic action, it has proven effective against *Campylobacter* and has shown sensitivity to it. Because of its effectiveness and observed sensitivity in antibiotic susceptibility testing, this antibiotic can be considered as one of the potential treatment options for the spotty liver syndrome in poultry. Due to the fact that ceftriaxone is used in cases of human infections and has limited application in the poultry industry, there are no comparable data available for broilers. Abd El‐Baky et al. reported resistance of *Campylobacter* strains isolated from human diarrheal cases to ceftriaxone [47]. The difference in results between Abd El‐Baky’s study and the current study may be due to the relationship between the host type and the antibiotic used in the target community. As ceftriaxone is not used in the poultry industry, it had good efficacy against the isolates in this study.

All of the isolates in the present study were MDR, which was similar to the results of Kalantar et al. in Iran [20]. In most cases, antibiotic treatment specifically targeting *Campylobacter* is not typically used in broiler poultry. However, the extensive and improper use of various antibiotic classes in poultry farming may contribute to the development of MDR strains, which, if transmitted to humans and causing disease, could complicate treatment. Recent studies have reported a rise in MDR isolates in poultry [2]. For example, Demiroğlu et al. reported 52.78% MDR isolates [25], and Khan et al. identified MDR in 97% of *C. jejuni* isolates in their respective studies [35]. Mutations in regulatory genes, particularly those affecting multidrug efflux pumps and resulting in increased gene expression, can result in bacterial resistance to multiple antibiotics and contribute to a rise in MDR isolates [46]. The varying prevalence rates of MDR *Campylobacter* in different flocks may be associated with the extensive use of various antibiotic families in the poultry industry [25]. In most cases, antibiotics are not used specifically for treating *Campylobacter* infections in broiler chickens. However, the heavy usage of different antibiotic classes in poultry farming can accelerate the development of MDR *Campylobacter* strains, which, if transferred to humans and causing disease, may complicate treatment options [25, 48].

The results obtained in the present study differ from those of other researchers in terms of antibiotic resistance patterns. This variation can be attributed to factors such as the excessive and indiscriminate use of antibiotics in humans, livestock, and poultry, including their use as growth promoters, the regulations governing antibiotic use in different countries, and the manner of monitoring their consumption [32, 41, 42]. As the results demonstrated, resistance was observed among the tested antibiotics that are commonly and routinely used in the poultry industry for treatment and prevention in Iran. All isolates also showed resistance to antibiotics considered the primary choices for treating human campylobacteriosis (erythromycin, tetracycline, and ciprofloxacin). The high antibiotic resistance observed in this study may support the hypothesis that the excessive use of antibiotics in broiler chicken farming in Isfahan Province has eliminated susceptible *Campylobacter* strains. Consequently, the isolated strains were those that had evolved by acquiring resistance genes against the antibiotics commonly used in the poultry industry. Since *Campylobacter* is transmitted via the fecal–oral route, widespread colonization by resistant strains in the future will threaten consumer health. Thus, it is recommended that, in addition to performing antibiograms before prescription, a review of antibiotic sales regulations, stricter monitoring of antibiotic usage, promotion of “No Antibiotics Ever” (NAE) farming, and improved slaughterhouse hygiene practices be considered. To further reduce the use of synthetic antibiotics as growth promoters and prophylactics, it is also suggested to use acidifiers, probiotics, and medicinal plant extracts with antibacterial properties.

## 5. Conclusion

The results of this study indicate the presence of *Campylobacter* (*jejuni* and *coli*) contamination in broiler flocks in Isfahan Province. The low presence of this bacterium may reflect the proper implementation of biosecurity measures in the farms of this region. The antibiotic lincomycin–spectinomycin is recommended as a potential treatment for *Campylobacter* infections.

## Author Contributions

Sajjad Abedini Chamgordani: conceptualization; investigation; visualization; project administration; and resources.

Mehdi Rezaei: conceptualization; investigation; funding acquisition; writing–original draft; writing–review and editing; visualization; validation; methodology; software; formal analysis; project administration; resources; data curation; and supervision.

Moslem Neyriz Naghadehi: data curation; supervision; formal analysis; validation; visualization; and writing–original draft.

## Funding

The authors declare that no funding was received for the conduct of this research or the preparation of this manuscript. This work was completed using the authors’ own resources.

## Conflicts of Interest

The authors declare no conflicts of interest.

## Data Availability

The data that support our findings are available from the corresponding author upon reasonable request.
